# Noncompliance with Ocular Hypertensive Treatment in Patients with Primary Open Angle Glaucoma among the Arab Population in Israel: A Cross-Sectional Descriptive Study

**DOI:** 10.1155/2013/405130

**Published:** 2013-07-01

**Authors:** Muhannad Masoud, Adi Sharabi-Nov, Joseph Pikkel

**Affiliations:** ^1^Department of Ophthalmology, Ziv Medical Center, P.O. Box 1008, 13100 Zefat, Israel; ^2^Faculty of Medicine, Bar Ilan University, 13101 Zefat, Israel

## Abstract

*Objective*. To evaluate the noncompliance treatment rates among primary open angle glaucoma (POAG) Arab patients in Israel and to verify the associated factors for noncompliance. *Patients and Methods*. A cross-sectional study took place using a questionnaire. Patients were initially interviewed and requested to answer a questionnaire. The questionnaire was developed based on a pilot test. Items included information about age, gender, number of prescribed drugs, and multiple reasons for noncompliance with drug therapy. *Setting*. Ophthalmologic HMO clinics, located in 3 Arab cities in the center of Israel. *Participants*. 400 Arab participants (197 men, 203 women) undergoing routine clinical care were recruited. *Results*. General rate of noncompliance, for both genders, was found to be 50%. Factors associated with nonadherence included inadequate knowledge (32%), underestimation of the disease severity (25.5%), and denial 15.5%. Compliance rates were unaffected by gender or number of prescribed drugs. Compliance was significantly higher in younger patients (age < 50) and in older patients (age > 80), 63% and 77%, respectively, (*P* < 0.05). *Conclusion(s)*. Noncompliance was found to be common among an Arab population in Israel, particularly between the ages of 50 and 80. Educational programs, improving patient-physician relationship, and personalizing treatment could provide means for improved adherence.

## 1. Introduction

Glaucoma is the second leading cause for blindness worldwide [1]. The prevalence of primary open angle glaucoma (POAG) is estimated to be between 1.1% and 3% in Western populations over the age of 40 years [1, 2]. The estimated number of POAG patients in Israel is 60,000. A large survey study including 10,000 Israeli participants demonstrated a similar distribution of the disease among various Israeli ethnicities, including Arabs [3].

In the literature, compliance is reported to vary from as low as 5% to as high as 80% [4]. However, limited information is available on this issue in the Middle East. Moreover, similar studies and information in the specific Israeli-Arab population are still lacking. The aim of this study is to estimate the degree, components, and determinants of noncompliance among POAG Arab patients in Israel and to verify associated factors for noncompliance. Glaucoma is insidious and disabling; the degree of interference with vision varies from imperceptible changes to complete blindness. Noncompliant patients exhibit a higher intraocular pressure and greater visual field loss [5]. Several authors have indicated that noncompliance is an important factor in blindness resulting from glaucoma [6–9]. 

## 2. Patients and Methods

A cross-sectional study took place throughout the year 2006. POAG patients attending out-patient eye clinics at the 3 cities, Tira, Taibi, and Qalanswa in Israel, were invited to join the study. Subjects were included if they were willing to participate, signed an informed consent, and fulfilled all of the following conditions:

- were a resident of one of the 3 cities (Tira, Taibi, or Qalnsawa) and from an Arab origin ethnicity;
- were diagnosed by an expert ophthalmologist as having POAG for at least 3 months prior to the interview; the diagnosis was based on glaucomatous cupping of the optic disc and a glaucomatous visual field loss, in one or both eyes, regardless of the intraocular pressure;
- were prescribed medications for glaucoma, in the past 3 months;
- had no mental or physical condition rendering them unable to participate in the study.

Exclusion criteria:

- evidence of angle closure glaucoma (ACG). ACG represents a group of ocular diseases with various causes that have a unique behavior and different treatment modalities (such as Laser Iridotomy);
- cataract surgery within 30 days prior to the study. Elevated intraocular pressure, following cataract surgery, occasionally requires a limited course of therapy to lower intraocular pressure perioperatively.

Four hundred consecutive patients constituted the population study.

The medical doctors at the vision clinics served as the interviewers in our study. They explained the purpose of the study to glaucoma patients in Arabic. All patients gave their written consent to participate in this survey.

A closed questionnaire, filled solely by medical doctors, was used to collect data during the interview. A detailed explanation was given by the medical staff for every question. The first section included general data of the patient, such as age and gender. The second section dealt with glaucoma treatment. The participants were asked three short questions:

- a list of prescribed glaucoma medications;
- the frequency of daily usage;
- the frequency of weekly and monthly usage.

Data retrieved, from both the out-patient clinic records and the questionnaires, were used to define compliance and noncompliance. Noncompliance was defined as missing at least one drop of medication per week and (or) the inability to accurately describe one's own medication regimen [9]. 

Subjects whom were considered as noncompliant were asked to choose one of the eight listed reasons to explain nonadherence (see the appendix). The noncompliance-related questions covered information regarding how to use eye drops, the importance of treatment and consequences following non-adherence, intolerance, and patient-physician interaction. Responses were grouped and analyzed into 5 categories by interviewing doctors (see the appendix).

## 3. Statistical Analysis

Multiple logistic regression analysis was performed to predict correlation between age and number of anti-IOP drugs. Logistic regression analysis was performed to predict compliance according to age. *T*-tests were carried out for gender and age. Statistical significance was considered positive if *P* < 0.05. In addition, chi-square tests were carried out in order to examine the relationships between gender and compliance and number of anti-IOP drugs.

## 4. Results

The overall noncompliance rate was 50%. Noncompliance rates were slightly higher among women, however, without statistical significance. Noncompliance rates were 47.3% among males and 52.7% among females. Mean age and SD among compliance patients group and noncompliance patients group were similar (Table 1). 

**Figure 1:**
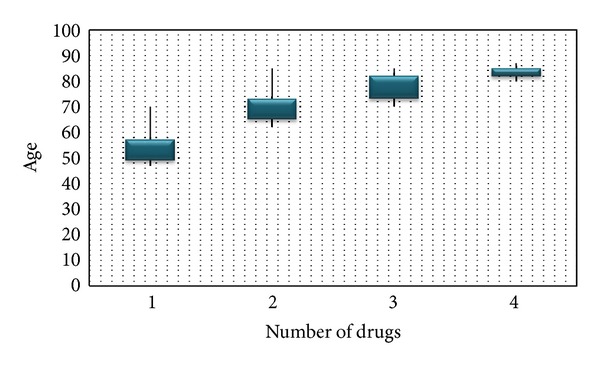
Box-plot distribution between age of the subjects and number of anti-IOP drugs.

We found a positive statistical significance in the correlation between age and number of prescribed drugs (*P* < 0.05). The older the patient, the more prescriptions for medication (Figure 1).

**Figure 2:**
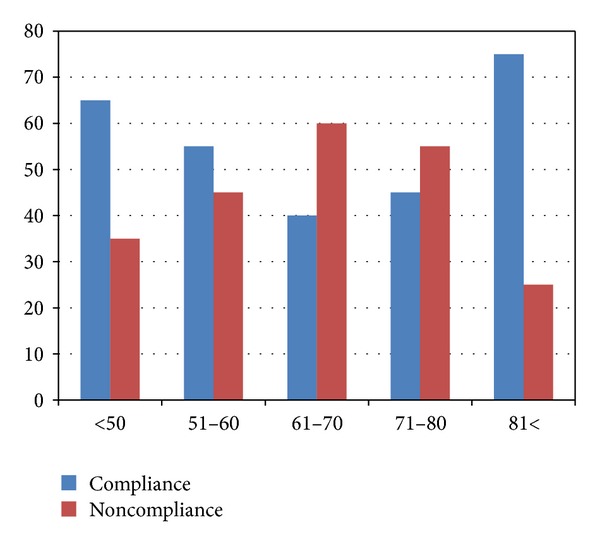
General compliance rates (white) and noncompliance (black) among subjects, according to age groups. *P* value represents the result of paired *t*-tests. Age categories <50 and >81 showed significant difference in compliance (*P* < 0.05).

Logistic regression analysis revealed that age cannot predict compliance (*P* > 0.05). Noncompliance rates were higher between ages 51 and 80 (Figure 2). Interestingly, the compliance was significantly higher among the two groups at either end of the curve, that is, younger than 50 years (77%) and older than 81 years (63%) (*P* < 0.05). No significant differences in compliance were found among the other age subgroups.

Though patients that were prescribed 3 drugs were slightly more noncompliant, the difference is statistically not significant and compliance rates were not influenced by gender or by number of anti-IOP drugs (Table 2).

**Figure 3:**
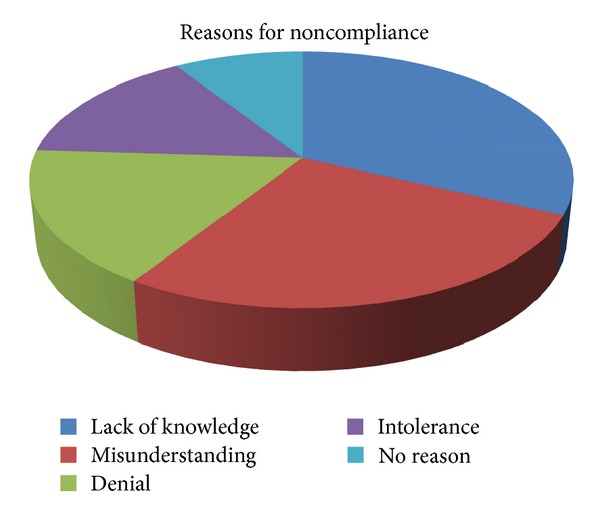
Reasons for noncompliance represented in 5 groups.

Figure 3 summarizes the main reasons given by the patients for noncompliance. Lack of knowledge (32%), misunderstanding (25.5%), and denial (15.5%) were the most common reasons indicated. In addition, 14% of the participants mentioned that they could not tolerate the drops, while 13% of the patients could not point to specific reasons for their noncompliance.

## 5. Discussion

The aim of this study was to find the noncompliance rates among COAG Israeli Arab patients and to try to define the reason for their noncompliance. The main findings of our survey are the relatively high noncompliance rates among COAG Israeli Arab patients. Our study has some limitations. In this study, medical doctors during the direct interview filled out the questionnaire. Under such conditions, a pleasing bias may occur, in which patients may tend to orally report better compliance. Furthermore, objectively measuring the effect of therapy, that is, the IOP, was not performed. However, in addition to the gathered data from the questionnaires, out-patient clinic records were retrieved and used together to define noncompliant patients, thus minimizing the aforementioned bias. 

Glaucoma patients who were not patients at outpatient eye clinics at Tira, Taibi, and Qalnsawa cities in Israel are not represented in this study. These unreported patients may be receiving treatment at private clinics or may have ceased to participate in their personal treatment plans. These patients may have characteristics different than patients coming for followup. Therefore, our study results represent only Israeli-Arab glaucoma patients treated in the outpatient clinics and may not represent all those suffering from glaucoma among the Israeli-Arab population. Furthermore, the study relies on patients' responses and involves health-care providers. Accordingly, it might be affected by a social pleasing bias. Moreover, better ways to measure disease progression compliance with medication than cross-sectional studies exist, implying that longitudinal studies are needed in the future to evaluate different levels of noncompliance.

Results of this study demonstrate that over 50% of the Israeli-Arab glaucoma patients are not compliant to their treatment plans. Lack of knowledge and misunderstanding are the major reasons given by patients explaining their non-adherence.

The reported noncompliance rates in Western populations and in the rest of Israel vary from 27.4% [10] to 42% [11] according to different studies. Various study designs and nonuniform compliance definitions explain this variability. The relatively high levels (50%) of noncompliance in the primarily Israeli-Arab population are consistent with many reported population-based studies in certain ethnic groups [12, 13]. For example, African Americans are not only more likely to develop POAG compared to white Americans, but also less compliant in their treatment plans [13]. Similarly, a recent study reported an even higher nonadherence rate among Oman glaucoma Arab patients (up to 75.2%) [14]. 

Purportedly, a higher rate of noncompliance among females (52.7% compared to males 47.2%) was observed in our survey. Yet, the differences are statistically insignificant, implying that gender per se is not associated with increased noncompliance [14, 15].

Figure 1 describes a positive association between age and number of anti-IOP drugs.

Compliance rate was unaffected by the number of drugs prescribed. Although overall noncompliance rates increased from 28.5% with once daily drug to 38% with 3x daily, this increment was statistically insignificant (Table 2). In addition, the frequency of instillation per day did not affect compliance significantly, although studies have shown that a single daily drug dose or combination is associated with better compliance rates [15, 16]. Granstrom and Norell have clearly shown that 4x daily regimen of pilocarpine was unrelated to low compliance [17]. However, noncompliance, in this study, is related to the patient's experience of side effects and/or negative attitudes to the treatment [17].

In our study, significantly higher rates of compliance were found among the two extreme age subgroups, that is, <50 years old and >80 years old (see Figure 2), 63% and 77%, respectively. Younger glaucoma patients are found to have a tendency to follow treatment regimens strictly probably due to proper perception of their disease and better instillation compared to older individuals [9]. As for the elderly, Gurwitz et al. found that elderly glaucoma patients started on multiple glaucoma medication were more adherent than those started on a single agent [18]. Therefore, in our older glaucoma group, a high probability of multiple medications might explain the high compliance rates. However, several studies clearly indicate higher prevalence rates of noncompliance among elderly, mainly due to improper installation of eye drops as well as other age-related medical illnesses, such as dementia and arthritis [9].

Clearly, a major determinant of compliance with medical treatment in any medical illness is the patient's awareness and understanding of his disease [19]. POAG usually produces few external signs, making the severity of the disease more difficult to perceive and possibly causing later complications, particularly because the only symptoms may be the side effects of the medical treatment prior to later more serious complications such as visual field loss [9]. Such facts could possibly explain the relatively higher rates of noncompliance in our patients.

Lack of knowledge and misunderstanding were the main factors cited for the >57% noncompliance rates in our patients. The association between compliance and awareness of the disease, its severity, treatment, and its late complications has been previously noted [9, 14, 20]. Furthermore, patients with higher levels of formal education are more accurate in describing their medication regimen [9]. In addition, better knowledge about glaucoma is associated positively with better compliance rates [14]. In a recent study in Oman, the knowledge about glaucoma was reported to be good in 23.8% of patients, who demonstrated higher compliance rates [14]. Kholdebarin et al. reported that over a third of glaucoma patients failed to use their ophthalmic medications properly, mainly due to lack of knowledge [9]. 

Many patients are in denial about their health problems, especially in asymptomatic diseases such as glaucoma. Not surprisingly, denial and intolerance comprised 30% of the noncompliant patients. In addition, the patient-physician interaction remains a cornerstone in improving compliance rates due to intolerance [7, 11, 15]. The physician should tailor the medication to the patient's lifestyle after careful investigation of the patient's routines such as sleeping, eating, and daily activity patterns [11, 14, 15]. Additionally, the physician must be aware of possible side effects and keenly seek them out, while always discussing various treatment options.

## 6. Conclusions

Noncompliance was found to be relatively common among Israeli-Arab COAG patients in both sexes. The overall noncompliance rate was 50% and was not influenced by gender or by number of anti-IOP drugs. Compliance was significantly higher among patients younger than 50 years (77%) and older than 81 years (63%). The most common reasons indicated for noncompliance were lack of knowledge, misunderstanding, and denial. Educating patients about their disease and its complications, using guiding brochures and DVDs, and improving the patient-physician relation by personalizing the treatment can plausibly improve compliance rates. Furthermore, patients' needs and knowledge should be taken into consideration in order to improve patients' compliances.

## Figures and Tables

**Table 1:** General characteristics of the participants according to age, gender, compliance, and noncompliance to glaucoma treatment.

Parameter	Compliance	Noncompliance	Total
Patients *N* (%)	200 (50)	200 (50)	400 (100)
Men *N* (%)	104 (52.8)	93 (47.2)	197
Women *N* (%)	96 (47.3)	107 (52.7)	203
Age (yrs) (mean ± SD)	68.6 ± 10.1	67.1 ± 10.1	67.8 ± 10.1

**Table 2:** General compliance according to number of drugs and gender.

Number of drugs	Gender	Compliance *N* (%)	Noncompliance *N* (%)	*P *
>3	M	5 (2.5)	3 (1.5)	NS
	F	7 (3.5)	7 (3.5)	
3	M	33 (16.5)	37 (18.5)	NS
	F	26 (13)	39 (19.5)	
2	M	32 (16)	26 (13)	NS
	F	33 (16.5)	31 (15.5)	
1	M	34 (17)	27 (13.5)	NS
	F	30 (15)	30 (15)	
Total to gender	M	**104 (53)**	**93 (47)**	NS
	F	**96 (47)**	**107 (53)**	

Total	—	200 (100)	200 (100)	—

## Appendix

### A. Questionnaire for the Glaucoma Noncompliance Study

#### A.1. Section One

Age
Gender
ID number.

#### A.2. Section Two

Glaucoma medications list

1. How many times during the day do you put eye drops in? Once, twice, three or four times a day?
2. Do you take your medications every day? (Yes/No)

After evaluating outpatient clinical medical records, and according to the above-listed data, the medical physician should classify the patient as compliant or noncompliant.

#### A.3. Section Three—Reasons for Noncompliance

(Only relevant to the noncompliant patients).

(1) What reason, from your perspective, explains most accurately why you don't take your medications properly?
  “I did not understand how to put the eye drops in.”
  “I didn't receive any medical explanation about my eye disease or about its treatment.”
  “The eye drops are useless.”
  “I put the eye drops in, once or twice, and then stopped using them.”
  “I am too busy and don't have spare time!”
  “I don't have any medical problems—I am not sick!”
  “I cannot stand the treatment.”
  “No particular reason.”

#### A.4. Later Analysis by the Interviewing Medical Doctor

(a) Lack of Knowledge
  “I did not understand how to put the eye drops in.”
  “I did not receive any medical explanation about my eye disease or about its treatment.”
(b) Misunderstanding
  “The eye drops are useless.”
  “I put the eye drops in, once or twice, and then stopped using them.”
(c) Denial
  “I am too busy and I don't have spare time!”
  “I don't have any medical problems—I am not sick!”
(d) Intolerance
  “I cannot stand the treatment.”
(e) No Reason
  “No particular reason.”

## References

[1] Quigley HA, Broman AT (2006). The number of people with glaucoma worldwide in 2010 and 2020. *British Journal of Ophthalmology*.

[2] Quigley HA (1996). Number of people with glaucoma worldwide. *British Journal of Ophthalmology*.

[3] Kurtz S, Goldenfeld M, Melamed S (2000). Early detection of glaucoma by a mobile unit—results from 10,000 examinees. *Harefuah*.

[4] Olthoff CMG, Schouten JSAG, van de Borne BW, Webers CAB (2005). Noncompliance with ocular hypotensive treatment in patients with glaucoma or ocular hypertension: an evidence-based review. *Ophthalmology*.

[5] Stewart WC, Chorak RP, Hunt HH, Sethuraman G (1993). Factors associated with visual loss in patients with advanced glaucomatous changes in the optic nerve head. *American Journal of Ophthalmology*.

[6] Musch DC, Gillespie BW, Niziol LM, Cashwell LF, Lichter PR (2008). Factors associated with intraocular pressure before and during 9 years of treatment in the Collaborative Initial Glaucoma Treatment Study. *Ophthalmology*.

[7] Suić SP, Cerovski B, Jukić T (2008). The role of patient compliance in the management of glaucoma. *Acta Medica Croatica*.

[8] Vorwerk C, Thelen U, Buchholz P, Kimmich F (2008). Treatment of glaucoma patients with insufficient intraocular pressure control: a survey of German ophthalmologists in private practice. *Current Medical Research and Opinion*.

[9] Kholdebarin R, Campbell RJ, Jin Y-P (2008). Multicenter study of compliance and drop administration in glaucoma. *Canadian Journal of Ophthalmology*.

[10] Stein JD, Ayyagari P, Sloan FA, Lee PP (2008). Rates of glaucoma medication utilization among persons with primary open-angle glaucoma, 1992 to 2002. *Ophthalmology*.

[11] MacKean JM, Elkington AR (1983). Compliance with treatment of patients with chronic open-angle glaucoma. *British Journal of Ophthalmology*.

[12] Leske MC, Connell AMS, Schachat AP, Hyman L (1994). The Barbados Eye Study: prevalence of open angle glaucoma. *Archives of Ophthalmology*.

[13] Tielsch JM, Katz J, Singh K (1991). A population-based evaluation of glaucoma screening: the Baltimore Eye Survey. *American Journal of Epidemiology*.

[14] Khandekar R, Shama ME-S, Mohammed AJ (2005). Noncompliance with medical treatment among glaucoma patients in Oman—a cross-sectional descriptive study. *Ophthalmic Epidemiology*.

[15] Patel SC, Spaeth GL (1995). Compliance in patients prescribed eyedrops for glaucoma. *Ophthalmic Surgery*.

[16] Buchan JC, Siddiqui S, Gilmour D (2007). Once daily drop regimes help reduce involuntary non-compliance. *Graefe’s Archive for Clinical and Experimental Ophthalmology*.

[17] Granstrom PA, Norell S (1983). Visual ability and drug regimen: relation to compliance with glaucoma therapy. *Acta Ophthalmologica*.

[18] Gurwitz JH, Glynn RJ, Monane M (1993). Treatment for glaucoma: adherence by the elderly. *American Journal of Public Health*.

[19] Sloan FA, Brown DS, Carlisle ES, Picone GA, Lee PP (2004). Monitoring visual status: why patients do or do not comply with practice guidelines. *Health Services Research*.

[20] Lau JTF, Lee V, Fan D, Lau M, Michon J (2002). Knowledge about cataract, glaucoma, and age related macular degeneration in the Hong Kong Chinese population. *British Journal of Ophthalmology*.

